# Alpha-lipoic acid attenuates cardiac fibrosis in Otsuka Long-Evans Tokushima Fatty rats

**DOI:** 10.1186/1475-2840-11-111

**Published:** 2012-09-19

**Authors:** Jung Eun Lee, Chin-ok Yi, Byeong Tak Jeon, Hyun Joo Shin, Soo Kyoung Kim, Tae Sik Jung, Jun Young Choi, Gu Seob Roh

**Affiliations:** 1 Department of Anatomy, Institute of Health Sciences, Gyeongsang National University School of Medicine, Jinju, Gyeongnam, Republic of Korea; 2 Department of Thoracic and Cardiovascular Surgery, Gyeongsang National University Hospital, Gyeongsang National University School of Medicine, Jinju, Gyeongnam, Republic of Korea; 3 Department of Internal Medicine, Gyeongsang National University Hospital, Gyeongsang National University School of Medicine, Jinju, Gyeongnam, Republic of Korea

**Keywords:** Alpha-lipoic acid, Cardiac fibrosis, OLETF rat

## Abstract

**Background:**

Hyperglycemia leads to cardiac oxidative stress and an imbalance in glucose homeostasis. Diabetic cardiomyopathy is characterised by cardiac hypertrophy and fibrosis. However, the underlying mechanisms of diabetic cardiomyopathy are not fully understood. This study aimed to investigate the effects of alpha-lipoic acid (ALA) on cardiac energy metabolism, antioxidant effect, and fibrosis in the hearts of Otsuka Long-Evans Tokushima fatty (OLETF) rats.

**Methods:**

Animals were separated into non-diabetic Long-Evans Tokushima Otsuka (LETO) rats and diabetes-prone OLETF rats with or without ALA (200 mg/kg/day) administration for 16 weeks. Diabetic cardiomyopathy was assessed by staining with Sirius Red. The effect of ALA on AMPK signalling, antioxidant enzymes, and fibrosis-related genes in the heart of OLETF rats were performed by Western blot analysis or immunohistochemistry.

**Results:**

Western blot analysis showed that cardiac adenosine monophosphate-activated kinase (AMPK) signalling was lower in OLETF rats than in LETO rats, and that ALA treatment increased the signalling in OLETF rats. Furthermore, the low antioxidant activity in OLETF rats was increased by ALA treatment. In addition to increased Sirius red staining of collagen deposits, transforming growth factor-β1 (TGF-β1) and connective tissue growth factor (CTGF) were expressed at higher levels in OLETF rat hearts than in LETO rat hearts, and the levels of these factors were decreased by ALA.

**Conclusions:**

ALA enhances AMPK signalling, antioxidant, and antifibrogenic effect. Theses findings suggest that ALA may have beneficial effects in the treatment of diabetic cardiomyopathy.

## Background

A constant rate of mitochondrial ATP synthesis and glucose uptake is necessary for the heart to continually contract [1]. Dysregulation of cardiac energy metabolism and insulin resistance causes morphological alterations in the myocardium [2]. In particular, previous studies have shown that perivascular and/or interstitial fibrosis are the most prominent myocardial structural changes in diabetic patients [3]. Despite the known relationship between energy metabolism and insulin resistance in the diabetic heart, the mechanism underlying the development of diabetic cardiomyopathy remains to be elucidated.

Adiponectin is an adipokine that has anti-diabetic and anti-atherogenic effects [4]. Hypoadiponectinemia leads to cardiac oxidative stress and dysregulation of glucose homeostasis [5]. Adiponectin is also synthesized and secreted by human and murine cardiomyocytes [6]. Adiponectin in insulin resistance correlates with activation of the adenosine monophosphate-activated kinase (AMPK) signalling pathway, which is implicated in fatty acid oxidation and glucose uptake. AMPK is a metabolic stress sensor or effector that controls energy homeostasis in the cell. AMPK is phosphorylated and activated by liver kinase B1 (LKB1) in response to an increase in the AMP/ATP ratio [7]. Activated AMPK phosphorylates and inactivates acetyl coenzyme A carboxylase (ACC), which is involved in fatty acid oxidation [8]. In adiponectin-deficient mice, diminished AMPK signalling in the heart is associated with increased cardiac hypertrophy [9].

Dysfunctional AMPK activity decreases antioxidant gene expression and induces inflammation and the production of oxidants [10]. An overabundance of oxidants is closely associated with insulin resistance. Overproduction of reactive oxygen species (ROS) is induced by hyperglycemia, dyslipidemia, advanced glycation end-products (AGEs), and lipid peroxides [11]. In particular, ROS production in mitochondria is increased in the diabetic heart, resulting in reduced cardiac energy metabolism [12].

Alpha-lipoic acid (ALA) was originally identified as an obligatory cofactor for mitochondrial α-ketoacid dehydrogenases and was found to play an important role in mitochondrial energy metabolism [13]. ALA enhances glucose utilization in isolated rat hearts [14]. Growing evidence suggests that ALA maintains the cellular antioxidant status by either enhancing or inducing the uptake of antioxidant enzymes [15]. ALA administration reduces aortic AGEs content, cardiac mitochondrial superoxide production, and insulin resistance in diabetic animal models [16].

Therefore, the purpose of this study was to investigate the effects of dietary ALA administration on the AMPK signalling pathway and on ROS associated with the development and progression of diabetic cardiomyopathy.

## Materials and methods

### Animals

Diabetes-prone male Otsuka Long-Evans Tokushima fatty (OLETF) rats (4 weeks old) and non-diabetic control Long-Evans Tokushima Otsuka (LETO) rats were obtained from the Otsuka Pharmaceutical Company (Tokushima, Japan) and maintained in the animal facility at Gyeongsang National University (Republic of Korea). All experiments were performed in accordance with the National Institutes of Health Guidelines on the Use of Laboratory Animals. The University Animal Care Committee for Animal Research of Gyeongsang National University approved the study protocol. LETO and OLETF rats were housed individually with an alternating 12-h light/dark cycle. OLETF rats (aged 12 weeks) were randomly separated into two groups (n = 9–10 per group) and were fed standard chow with or without ALA (200 mg/kg/day, Bukwang Pharmaceutical Company, Seoul, South Korea) for 16 weeks. LETO rats were fed standard chow without ALA. All rats were weighed immediately before sacrifice at 28 weeks of age.

### Tissue collection and sample preparation

For tissue analysis, rats were anesthetized with Zoletil (5 mg/kg, Virbac Laboratories, Carros, France) and then perfused transcardially with heparinized saline followed by 4% paraformaldehyde in 0.1 M phosphate buffered saline (PBS). The hearts were fixed with the same reagent for 12 h at 4°C. The samples were then processed for paraffin embedding, and 5 μm-thick sections were cut. Sections were stained with hematoxylin and eosin (H&E). The sections were visualized under a BX51 light microscope (Olympus, Tokyo, Japan), and digital images were captured and documented.

### Sirius red staining

Sirius red staining is commonly used to identify collagens. To determine cardiac collagen accumulation, deparaffinized heart sections were stained with Weigert’s hematoxylin (Sigma-Aldrich, MO, USA) for 8 min, washed, and restained with picro-sirius red (Sigma) for 1 h and washed. Sections were dehydrated through graded alcohols, cleared in xylene, covered with a coverslip, and sealed with Permount (Sigma).

### Sircol collagen assay

The Sircol collagen assay is a dye-binding method designed for the analysis of acid and pepsin-soluble collagens, which are newly synthesized during inflammation and wound healing. The heart tissues were frozen in liquid nitrogen and stored at -80°C prior to the assay. The collagen concentration was analysed using a Sircol assay kit (Bioclor Ltd., Northern Ireland, UK) according to the instructions provided by the manufacturer. A standard curve was derived and the collagen content of the sample was calculated.

### Immunohistochemistry

Deparaffinized heart sections were placed in a solution of 0.3% H_2_O_2_ for 10 min. After washing, sections were treated with diluted blocking goat serum for 20 min. Slides were incubated overnight at 4°C in a humidified chamber with anti-mouse-Cu/Zn-superoxide dismutase (SOD) (1:100, Santa Cruz Biotechnology, USA) diluted in blocking serum. After washing three times with 0.1 M PBS, sections were incubated for 1 h at room temperature with a secondary antibody (1:200). After washing, sections were incubated in avidin-biotin-peroxidase complex solution (ABC solution, Vector Laboratories, Burlingame, CA, USA). Sections were developed with 0.05% diaminobenzidine (DAB, Sigma) containing 0.05% H_2_O_2_ and were dehydrated through graded alcohols, cleared in xylene, covered with a coverslip, and sealed with Permount (Sigma). Sections were visualized under a BX51 light microscope (Olympus). For immunostaining of collagen tissue growth factor (CTGF), heart sections were incubated with the rabbit anti-rat CTGF (1:500, Abcam, Cambridge, MA, USA) overnight at 4°C. Sections were incubated with AlexaFluor 594-conjugated donkey anti-rabbit antibody (1:1,000, Invitrogen, Carlsbad, CA, USA). Fluorescence was visualized under a confocal microscope (FV-1000, Olympus).

### Cytosolic and nuclear fraction

For cytosolic and nuclear fractions, the hearts were promptly excised and placed in ice-cold PBS. After chopping in ice-cold lysis buffer (10 mM HEPES-KOH [pH7.9], 1.5 mM MgCl_2_, 10 mM KCl, 1 μg/ml aprotinin, 3 μg/ml pepstatin, 0.5 μg/ml leupeptin, 0.2 mM PMSF, 0.5 mM DTT), the hearts were homogenized. The fractions of heart were prepared according to Andrews and Faller [17]**.**

### Membrane fractionation

The hearts were promptly excised and placed in ice-cold PBS. After chopping in ice-cold hypertonic lysis buffer (10 mM Tris, 10 mM NaCl, 3 mM MgCl2, 1 mM sodium vanadate, 5 μg/ml aprotinin, 3 μg/ml pepstatin, 5 μg/ml leupeptin, 1 mM EDTA, 1mM DTT), the hearts were homogenized. Homogenates were centrifuged at 12,500 × *g* for 15 min. The resulting pellet were resuspended in 1% Triton lysis buffer and centrifuged at 12,500 × *g* for 15 min.

### Western blot analysis

For total heart extracts, frozen hearts were homogenized in a T-PER tissue protein extraction reagent (Thermo scientitic, IL, USA) containing Halt protease inhibitor cocktail (Thermo scientitic). The following antibodies were used: LKB1 (Wako Pure Chemical Company, Osaka, Japan); phospho-AMPK, AMPK, phospho-acetyl-CoA carboxylase (ACC), ACC, Sterol regulatory element-binding protein-1 (SREBP-1, BD Biosciences, CA, USA), glucose transporter 4 (GLUT4, Cell Signaling Technology, Danvers, MA, USA), receptor for advanced glycosylation end products (RAGE), heme oxygenase-1 (HO-1), Cu/Zn-SOD, and transforming growth factor-β1 (TGF-β1) (all from Santa Cruz Biotechnology). The membranes were probed with each antibody or α-tubulin antibody (Sigma) and visualized using an enhanced chemiluminescence substrate (Pierce, Rockford, IL, USA). The Multi-Gauge V 3.0 image analysis program (Fujifilm, Tokyo, Japan) was used to measure band density.

### Statistical analysis

Differences between LETO, OLETF, and OLETF rats following ALA administration were determined with one-way ANOVA, followed by Bonferroni post-hoc analysis. Values are expressed as the mean ± standard error of the mean (SEM). A *p* value < 0.05 was considered statistically significant.

## Results

### Effect of ALA on heart and body weight of OLETF rats

OLETF rats were fed ALA for 16 weeks. Without ALA treatment, the body weight of OLETF rats at 28 weeks was significantly higher than that of LETO rats (*p* < 0.05) (Table 1). However, ALA caused a significant reduction in the body weight of OLETF rats (*p* < 0.05). Whole heart weights were measured at the time of sacrifice, and the heart/body weight ratio was calculated for each group. Although OLETF heart weights were higher than those of LETO rats, the heart/body weight ratio was significantly lower in OLETF rats without ALA treatment than in OLETF rats with ALA treatment (*p* < 0.05).

**Table 1:** Body and heart weight in OLETF rats with or without ALA treatment

	**LETO**	**OLETF**	**OLETF + ALA**
**Body weight (g)**	538.55 ± 8.63	636.40 ± 15.55^*^	518.54 ± 14.92^†^
**Heart weight (g)**	1.36 ± 0.03	1.52 ± 0.02^*^	1.43 ± 0.03^†^
**Heart/body weight (ratio)**	2.73 ± 0.05	2.52 ± 0.09^*^	3.05 ± 0.05^†^

Significance: ^*^P < 0.05. vs. LETO rats, ^†^*P* < 0.05. vs. OLETF rats.

### Effect of ALA on cardiac AMPK signalling pathway in OLETF rats

**Figure 1:**
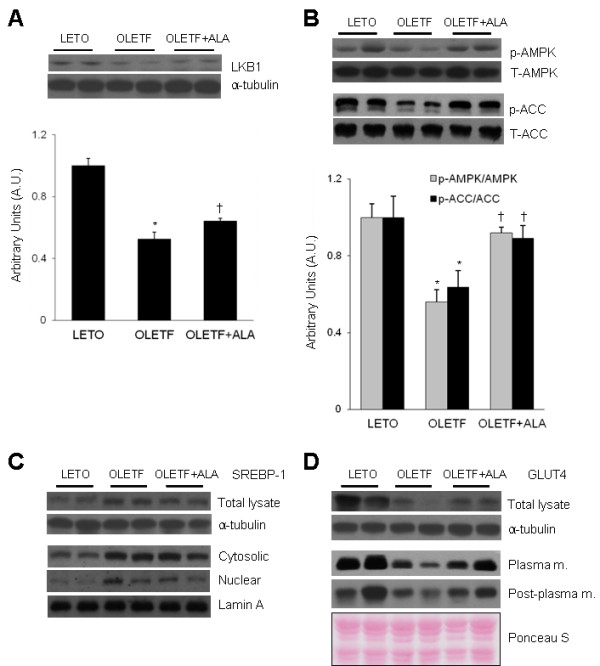
**Effect of ALA on the cardiac AMPK signalling pathway in OLETF rats. **(**A**) Representative Western blots of LKB1. Quantification of cardiac LKB1 by Western blot analysis. (**B**) Western blot showing total (T) and phosphorylated (p) forms of AMPK and ACC in the hearts from each group. Quantification of the phosphorylation of each protein on Western blots. The densitometry value for each phospho-protein was normalized against total protein and the values are presented as arbitrary units (A.U.). Data are presented as the mean ± SEM. *p < 0.05 vs. LETO rats; †*p* < 0.05 vs. OLETF rats. (**C**) A Western blot showing the level of cardiac SREBP1 in each group. The total, cytosolic, and nuclear lysates were prepared from heart tissues. a-tubulin and lamin A were used as the control to verify identical protein loading. (**D**) A western blot showing the level of cardiac GLUT4 in each group. The total lysate, plasma membrane, and post-plasma membrane fraction were prepared from heart tissues. a-tubulin and Ponceau S dye staining was used as the control to verify identical protein loading.

To determine the effect of ALA on cardiac LKB1 expression, Western blot analysis was performed (Figure 1A). Levels of cardiac LKB1 expression were significantly lower in OLETF rats than in LETO rats (*p* < 0.05). However, ALA significantly increased LKB1 expression in OLETF rats (*p* < 0.05). The effects of ALA on the phosphorylation of AMPK and ACC expression were then evaluated (Figure 1B). Western blot analysis showed that the levels of cardiac phospho (p)-AMPK and p-ACC in OLETF rats were lower than in LETO rats and that they increased after ALA administration (*p* < 0.05). To investigate the dynamics of the downstream AMPK signalling pathways in the heart, Western blot analysis of SREBP1 and GLUT4 expression was performed (Figure 1C and D). Compared with LETO rats, western blot revealed that there is an increase of precursor segment of SREBP-1 expression in both total and cytosolic lysates OLETF rats approximately to 1.42 and 4.51 times, respectively (Figure 1C). Also, mature segment of SREBP-1 was increased in OLETF rats compared with LETO rats. However, ALA treatment attenuated SREBP-1 expression in total and nuclear lysates from the heart of OLETF rats (1.24 and 2.76 times, respectively, *p* < 0.05). In the heart tissues, the GLUT4 levels in all the lysates were decreased in OLETF rats compared with LETO rats (Figure 1D). ALA treatment increased GLUT4 translocation from intracellular sites to the plasma membrane.

### Effect of ALA on cardiac RAGE expression in OLETF rats

**Figure 2:**
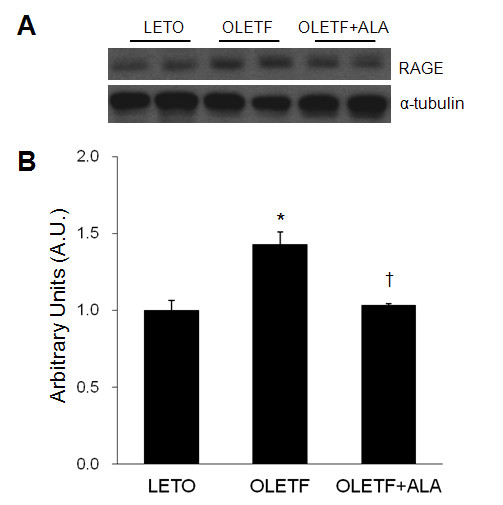
**Effect of ALA on cardiac RAGE expression in OLETF rats. **(**A**) Western blot showing the level of cardiac RAGE in each group. (**B**) Quantification of cardiac RAGE by Western blot analysis. Densitometry values of RAGE protein were normalized to that of α-tubulin and are presented as arbitrary units (A.U.) relative to LETO expression levels. Data are presented as the mean ± SEM. *p < 0.05 vs. LETO rats; †*p* < 0.05 vs. OLETF rats.

The effect of ALA on cardiac RAGE expression was evaluated in OLETF rats by Western blot analysis (Figure 2A). Cardiac RAGE expression was significantly higher in OLETF rats than in LETO rats, and ALA significantly decreased RAGE expression in OLETF rats (*p* < 0.05) (Figure 2B).

### Effect of ALA on cardiac HO-1 and Cu/Zn-SOD expression in OLETF rats

**Figure 3:**
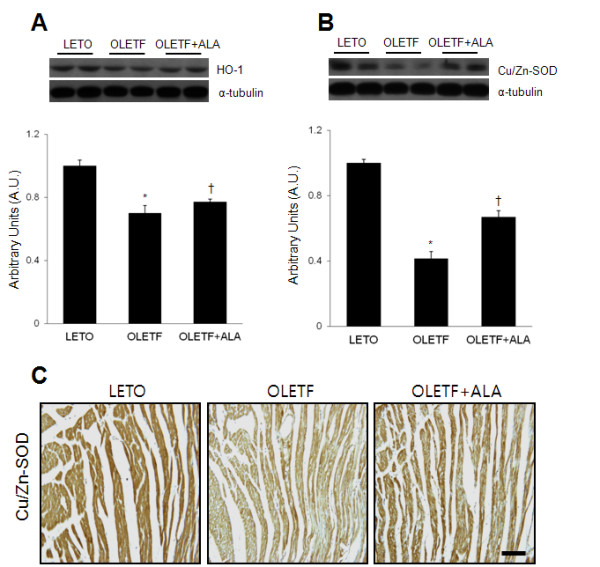
**Effect of ALA on cardiac HO-1 and Cu/Zn-SOD expression in OLETF rats. **(**A**) Western blot showing the level of cardiac HO-1 in each group. Quantification of cardiac HO-1 by Western blot analysis. (**B**) Western blot showing the level of cardiac Cu/Zn-SOD in each group. Quantification of cardiac Cu/Zn-SOD by Western blot analysis. The densitometry value of each protein was normalized to that of α-tubulin and the values are presented as arbitrary units (A.U.) relative to LETO expression levels. Data are presented as the mean ± SEM. *p < 0.05 vs. LETO rats; †*p* < 0.05 vs. OLETF rats. (**C**) Representative micrographs of immunostained cardiac Cu/Zn-SOD in each group. Scale bar = 100 μm.

To investigate the effect of ALA on antioxidant enzyme activity in response to oxidative stress in OLETF rat hearts, Western blot analysis of HO-1 and Cu/Zn-SOD expression and immunohistochemistry of Cu/Zn-SOD were performed (Figure 3). Western blot analysis revealed that cardiac HO-1 and Cu/Zn-SOD expression levels were significantly lower in OLETF rats than in LETO rats (Figure 3A and B). However, the expression of both proteins was significantly increased in OLETF rats by ALA treatment (*p* < 0.05). Immunohistochemistry showed that Cu/Zn-SOD-positive cells were distributed throughout the cardiomyocytes of LETO rats and OLETF rats with ALA treatment (Figure 3C). However, without ALA treatment, Cu/Zn-SOD-positive cells were stained more weakly in OLETF rats.

### Effect of ALA on collagen accumulation in OLETF rat hearts

**Figure 4:**
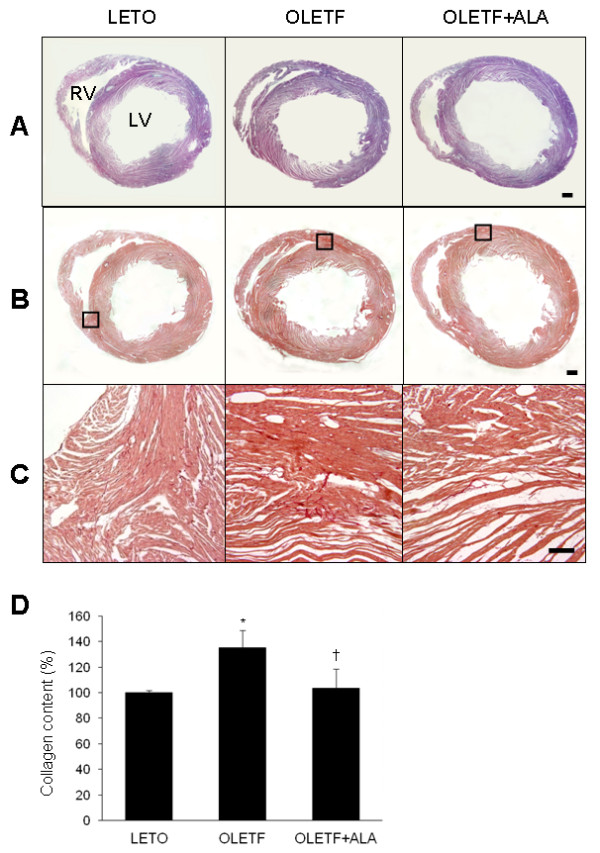
**Effect of ALA on cardiac collagen deposition in OLETF rats. **Representative micrographs of H&E-stained (**A**) and Sirius red-stained (**B**) heart sections from each group. The black lined-box (in B) shows a high-magnification micrograph (**C**) of Sirius red-stained sections. Scale bar = 1000 μm (200 μm in C). (**D**) Sircol collagen assay quantifying soluble collagen in the hearts of LETO and OLETF rats with or without ALA. Data are presented as the mean ± SEM. *p < 0.05 vs. LETO rats; †*p* < 0.05 vs. OLETF rats.

To examine the effects of ALA on cardiac morphology in 28-week-old OLETF rats, H&E staining was performed (Figure 4A). No significant morphological changes were observed between LETO and OLETF rats. Sirius red-stained collagen deposits were observed in the left ventricles (LV) of OLETF rat hearts (Figure 4B and C). However, ALA treatment reduced Sirius red-stained collagen deposition. The effect of ALA on collagen synthesis in OLETF rat hearts was confirmed using the Sircol collagen assay (Figure 4D). As observed with Sirius red staining, OLETF rats had significantly more soluble collagen than LETO rats (*p* < 0.05). After ALA administration, a significant decrease in the quantity of collagen was observed (*p* < 0.05).

### Effect of ALA on TGF-β1 and CTGF expression in OLETF rat hearts

**Figure 5:**
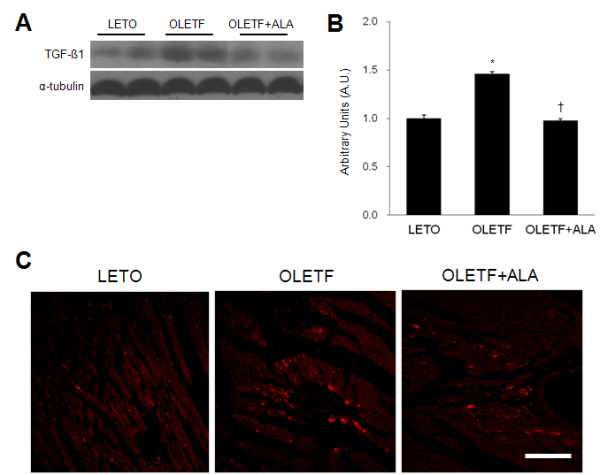
**Effect of ALA on cardiac TGF-β1 and CTGF expression in OLETF rats. **(**A**) A Western blot showing the level of cardiac TGF-β1 in each group. (**B**) Quantification of cardiac TGF-β1 by Western blot analysis. The densitometry value of each protein was normalized to that of α-tubulin and the values are presented as arbitrary units (A.U.) relative to LETO expression levels. Data are presented as the mean ± SEM. *p < 0.05 vs. LETO rats; †*p* < 0.05 vs. OLETF rats. (**C**) Representative micrographs of immunostained CTGF in the hearts from each group. Scale bar = 100 μm.

The effect of ALA on cardiac TGF-β1 and CTGF expression was evaluated in OLETF rats by Western blot and immunofluorescence analyses, respectively (Figure 5). Cardiac TGF-β1 expression was significantly higher in OLETF rats than in LETO rats (*p* < 0.05), and ALA treatment significantly decreased TGF-β1 expression in OLETF rats (*p* < 0.05) (Figure 5A and B). CTGF-positive cells were distributed throughout the cardiomyocytes in OLETF rats (Figure 5C). However, CTGF staining was weak in LETO rats and OLETF rats treated with ALA.

## Discussion

The results of this study demonstrate that ALA stimulates the AMPK signalling pathway and attenuates cardiac fibrosis in OLETF rats. ALA increased phosphorylation of AMPK and ACC, decreased SREBP-1, and increased GLUT4 translocation in the hearts of OLETF rats. Furthermore, ALA decreased RAGE, TGF-β1, and CTGF expression by increasing the activity of antioxidant enzymes, such as HO-1 and Cu/Zn-SOD. Thus, the finding that ALA attenuates collagen accumulation in the heart of diabetes-prone OLETF rats by improving cardiac lipid metabolism and antioxidant activity indicates that chronic dietary administration of ALA to pre-diabetic OLETF rats could prevent diabetic cardiomyopathy.

A previous study showed that high dose of ALA reduces body weight in obese humans [18], ALA treatment is associated with body fat loss, which it mediates by suppressing AMPK activity in the hypothalamus [19]. This effect was attributed to the appetite-suppressing properties of ALA and to increased energy expenditure. However, the mechanism by which ALA induces body weight loss is poorly understood. In streptozotocin (STZ)-induced animal model, ALA did not attenuate the weight loss in control rats [20]. Previous studies showed that 180 mg/kg/day of 24-months ALA supplementation in Sprague-Dawley rats showed no serious adverse effects in hematology, biochemistry, organ gross pathology, and neoplasm [21]. Thus, our study did not contain ALA-treated LETO rats. Although recent scientific studies emphasizes that obesity is major risk factor for diabetic cardiomyopathy, the relationship between obesity and heart function is not completely known. However, pharmacologic strategies for contribution of weight loss and prevention of weight gain are reported [22,23]. Recent report showed that impaired left ventricular ejection fraction (LVEF), enhanced LV remodeling, inflammation, and fibrosis were reversed by obesity reduction in obese mice [24]. These findings suggested the important role of obesity in tissue damage to the myocardium other than those related to diabetic coronary artery diseases. In addition, myocardial apoptosis, fibrosis, and anti-oxidant biomarkers in LV myocardium were significantly suppressed in obese mice and reversed in obese mice after reduction of body weight. In our study, ALA treatment significantly attenuated heart and body weight in OLETF rats. Although the heart weight of OLETF rats was higher than that of LETO rats, the heart to body weight ratio was not increased. This does not necessarily mean that a high heart weight is closely associated with diabetic cardiac hypertrophy. ALA could have significantly contributed to the reduction in body weight with affecting cardiac inflammation and fibrosis in this pre-diabetic animal model. Thus, our findings suggest that pharmacological treatment could at least partially support why diabetic cardiomyopathy was enhanced after weight reduction.

### Effects of ALA administration on adiponectin-AMPK signalling pathways in the diabetic heart

Adiponectin increases insulin sensitivity by increasing fatty acid oxidation, resulting in reduced circulating fatty acid levels and reduced triglyceride (TG) content in muscle [25]. Energy homeostasis is vital for continuous cardiac pumping activity, and adiponectin controls energy homeostasis by modifying through glucose uptake [26]. In our previous studies, serum adiponectin was shown to be expressed at lower levels in OLETF rats than in LETO rats, and ALA increased adiponectin levels in OLETF rats. [27]. AMPK is phosphorylated and activated by its upstream kinase, LKB1, and both are conserved serine/threonine kinases that regulate metabolism [28]. In this study, diabetes-prone OLETF rats had low cardiac LKB1 expression, which was increased by ALA administration. This result is consistent with the report that obese insulin-resistant Zucker rats have decreased LKB1 content in muscle [29]. Moreover, the lower expression of LKB1 in the heart correlated closely with lower AMPK/ACC signalling pathway activity. These results support a role for ALA in promoting the effects of SIRT1 activation and LKB1-AMPK signalling on insulin sensitivity [30,31]. SREBP1, which is negatively regulated by AMPK, is a major regulator of fatty acid synthesis [32]. Consistent with the observation that AMPK inhibits lipogenesis by reducing SREBP1 expression and by activating glucose uptake via GLUT4 upregulation [27,33], ALA reversed the increase in the levels of SREBP1 and decreased the levels of GLUT4 in OLETF rat hearts. In our previous study, we also confirmed the effect of ALA on SREBP1 and GLUT4 expression in non-alcoholic fatty liver disease of OLETF rats [27]. SREBP1 expression is significantly higher in nonalcoholic fatty liver disease than in control animals [34]. ALA reduces circulating free fatty acids (FFA) and TG levels by reducing lipid accumulation in non-adipose tissue as well as in adipose tissue [27,35]. In addition, our study confirms that ALA may contribute to inhibit the proteolytic cleavage and nuclear translocation of SREBP-1 in the heart of diabetic OLETF rats. This finding is in agreement with the results reported by Hao et al. [36] that high glucose increase lipogenesis by increasing precursor and mature (cleaved form) segment of SREBP-1 in renal tubular cells and HKC cells. The roles of cardiac glucose uptake and insulin action have been demonstrated in mice with cardiac-specific ablation of GLUT4, which developed cardiac hypertrophy resembling that of the diabetic heart [37]. In OLETF rats, caloric restriction improves insulin resistance in association with increased adipocyte-specific GLUT4 expression. It has been reported that impairment of glucose uptake in obesity is closely associated with the reduction of cellular GLUT4 content and translocation into plasma membrane [38,39]. Our study shows that the protein expression of both total lysates and plasma membrane is decreased, indicating that glucose metabolism would be reduced in the heart of OLETF rats. However, ALA enhanced cellular GLUT4 contents and translocation. This finding is in agreement with the results reported by Park et al. [38] and Guo et al. [33] that caloric restriction or telmisartan reduces insulin resistance by improving GLUT4 gene expression and GLUT4 translocation to the plasma membrane. Penumathsa et al. [40] also demonstrated that the antioxidant resveratrol enhances GLUT4 translocation in the STZ-induced diabetic heart. This suggests that obesity-induced cardiac dysfunction may be attributable to chronic alterations in cardiac glucose and lipid metabolism and in the levels of circulating adipokines, including adiponectin.

### ALA has antioxidant and anti-inflammatory effects in the diabetic heart

In addition to cardiac dysfunction caused by energy disturbances and oxidative stress, an association between the deleterious effects of AGEs and diabetic vascular complications has been suggested in many human studies [41]. Kuhla et al. [42] suggested that targeting the AGE/RAGE interaction with an inhibitor of RAGE may be of therapeutic value in oxidative stress-induced hepatic inflammation. Our results showed that ALA inhibited increased cardiac RAGE expression in OLETF rats. These data indicate that the oxidative stress-dependent AGE/RAGE interaction may be regulated by the antioxidant ALA. In this study, ALA increased the antioxidant activity in OLETF rats. Ogborne et al. [43] first reported that ALA increases HO-1 expression in human monocytic THP-1 cells. In Zucker diabetic fat rats, upregulation of HO-1 activity induced by protoporphyrin increased adiponectin levels and improved insulin sensitivity by increasing AMPK phosphorylation, and decreased adipose tissue volumes [44]. In addition to HO-1 expression, Cu/Zn-SOD expression, which was reduced in OLETF rat hearts, was increased by ALA treatment. The downregulation of antioxidant enzymes, including HO-1 and Cu/Zn-SOD, under conditions of chronic obesity or insulin resistance-induced oxidative stress, may promote the progression of diabetic cardiomyopathy.

### ALA has antifibrogenic effects in the diabetic cardiomyopathy

Diabetes-induced cardiac fibrosis is a major risk factor for the progression of diabetic cardiomyopathy, which can result in cardiac cell death, fibrosis, and endothelial dysfunction [45,46]. Guo et al. [6] demonstrated that decreased plasma adiponectin levels may contribute to myocardial hypertrophy in STZ-induced diabetic rats. Furthermore, adiponectin supplementation improved concentric cardiac hypertrophy in adiponectin-deficient mice [9]. Recent study showed that recombinant human granulocyte-colony stimulating factor (G-CSF) ameliorates cardiac diastolic dysfunction and fibrosis in OLETF rats [47]. Although cardiac hypertrophy was not detected in the OLETF rat model used in this study, recent study showed that ALA (100 mg/kg/day) reversed impairment of systolic function in STZ-treated diabetic rats compared to controls [21]. Indeed, diabetic heart disease is associated with increased interstitial fibrosis, which is caused by collagen accumulation via an increase in the level of type III collagen [48]. Consistent with the observation that ALA ameliorates cardiac fibrosis in STZ-induced diabetes [20], Sirius red staining also showed that ALA inhibited collagen accumulation in marginal regions between the right and left ventricles in OLETF rat hearts. Thus this data suggest that comparing STZ-induced diabetic rats, diabetes prone-OLETF rats induce mild diabetic cardiomyopathy. TGF-β1 is a key factor in the formation of fibrosis, which results from collagen deposition. During cardiac pathology, TGF-β1 is expressed at high levels in the heart [49]. CTGF, which is a potent profibrotic factor, induces the accumulation of collagen by stimulating cardiac fibroblasts in response to TGF-β1 [50]. Western blot analysis of TGF-β1 expression and immunofluorescence analysis of CTGF expression showed that CTGF-positive cell number was reduced by ALA treatment. Our results support the hypothesis that hyperglycemia induces changes in cardiac structure via the generation of AGEs and ROS, and via TGF-β1 stimulation [51].

## Conclusion

Collectively, these data demonstrate that ALA enhances the AMPK/ACC/SREBP1/GLUT4 signalling pathway, inhibits RAGE expression, reduces oxidative stress, and prevents myocardial fibrosis in OLETF rats. Thus, this study suggests that hyperglycemia and obesity exacerbate diabetic cardiomyopathy by inducing cardiac fibrosis and dysregulation of energy homeostasis.

## Abbreviations

ACC: Acetyl-CoA carboxylase; AGE: Advanced glycation end-products; AMPK: Adenosine monophosphate-activated kinase; ALA: Alpha-lipoic acid; CTGF: Connective tissue growth factor; FFA: Free fatty acids; GLUT4: Glucose transporter 4; HO-1: Heme oxygenase-1; LETO: Long-Evans Tokushima Otsuka; LKB1: Liver kinase B1; OLETF: Otsuka Long-Evans Tokushima fatty; RAGE: Receptor for advanced glycosylation end products; ROS: Reactive oxygen species; SREBP-1: Sterol regulatory element-binding protein-1; SOD: Superoxide dismutase; TG: Triglyceride; TGF-β1: Transforming growth factor-β1.

## Competing interests

All authors declare that they have no competing interests.

## Authors’ contributions

We thank all other investigators. JEL researched data, contributed to discussion, wrote the manuscript. CY, BTJ, and HJS researched data. SKK, TSJ, and JYC contributed to discussion and reviewed. GSR researched data, contributed to discussion, wrote the manuscript, and reviewed and edited the manuscript. All authors read and approved the final manuscript.
